# An Enterovirus-Like RNA Construct for Colon Cancer Suicide Gene Therapy

**DOI:** 10.7508/ibj.2015.03.001

**Published:** 2015-07

**Authors:** Mahsa Rasekhian, Ladan Teimoori-Toolabi, Safieh Amini, Kayhan Azadmanesh

**Affiliations:** 1 *Dept. of Virology, Pasteur Institute of Iran, Tehran, Iran; *; 2 *Dept. of Molecular Medicine, Pasteur Institute of Iran, Tehran, Iran;*; 3 *Dept. of Hepatitis and HIV, Pasteur Institute of Iran, Tehran, Iran*

**Keywords:** Thymidine kinase, Polio 3’NCR, miR-143

## Abstract

**Background:**

In gene therapy, the use of RNA molecules as therapeutic agents has shown advantages over plasmid DNA, including higher levels of safety. However, transient nature of RNA has been a major obstacle in application of RNA in gene therapy.

**Methods:**

Here, we used the internal ribosomal entry site of encephalomyocarditis virus and the 3’ non-translated region of Poliovirus to design an enterovirus-like RNA for the expression of a reporter gene (enhanced green fluorescent protein) and a suicide gene (thymidine kinase of herpes simplex virus). The expression of these genes was evaluated by flow cytometry and cytotoxicity assay in human colorectal adenocarcinoma cell line (SW480). We then armed RNA molecules with a target sequence for hsa-miR-143 to regulate their expression by microRNA (miRNA) mimics.

**Results:**

The results showed effective expression of both genes by Entrovirus-like RNA constructs. The data also showed that the restoration of hsa-miR-143 expression in SW480 leads to a significant translation repression of the introduced reporter and suicide genes.

**Conclusion:**

Collectively, our data suggest the potential use of Entrovirus-like RNA molecules in suicide gene therapy. Additionally, as a consequence of the possible downregulated miRNA expression in cancerous tissues, a decreased expression of gene therapy constructs armed with target sequences for such miRNA in cancer tissue is expected.

## INTRODUCTION

Despite the significant progress in cancer therapy, the burden of the disease remains high, requiring new strategies for treatment [1]. Suicide gene therapy, which involves the conversion of a non-toxic pro-drug into a cytotoxic compound by intracellular delivery of an enzyme-coding sequence, is one of the several promising approaches in cancer therapy [2, 3]. However, the possibility of integration into the host genome and aberrant expression of the transgene has raised the safety issues associated with the use of DNA-based carriers. Moreover, the expression of suicide genes in non-targeted tissues restricts the applicability of this method [4]. For amelioration of the safety concerns related to the use of DNA, messenger RNA (mRNA) has been proposed as a vehicle for gene delivery [5]. The possibility of direct delivery of mRNA to the cytoplasm, hence avoiding the nuclear membrane, leads to a more rapid expression of the transgene compared to a DNA delivery molecule [6]. Additionally, the risk for integration of the transgene into the host genome is defeated by replacing DNA molecules with RNA counterparts. Hence, the expression of foreign genetic information for a limited period of time is guaranteed when using RNA instead of DNA. However, the susceptibility of RNA molecules to endogenous nucleases and their short half-life have limited their use in gene therapy. In this regard, the *in vitro* chemical modifications can increase the resistance of these molecules to degradation by nuclease [7, 8]. Moreover, it has been shown that secondary structures found in 5′ and 3′ untranslated regions (UTR) of RNA increase the half-life of these molecules through interaction with cellular factors such as RNA-binding proteins [9].

Using the thymidine kinase gene of herpes simplex virus type I (HSV1-TK) in conjunction with inactive ganciclovir (GCV) as a pro-drug is the most promising approach in cancer treatment; however, the non-specific expression of the gene in non-cancerous cells has seriously limited its application [10]. Targeted delivery of the toxic genes by viral vectors has been practiced using tissue-specific promoters. However, the leaky expression of toxic gene in normal cells has made the use of mRNA as a direct source of gene product more attractive [11]. Target specificity of the suicide gene-expressing mRNA could be achieved using the tissue-specific microRNA (miRNA) [12]. This group of small, non-coding RNA acts in various regulatory systems, including post-translational regulation of gene expression. Mature miRNA is recruited to the RNA-induced silencing complex and interacts with its target mRNA. The interaction of miRNA with the 3′UTR of protein-coding mRNA is considered as the main mechanism of gene silencing. This usually leads to a decrease in protein expression either by mRNA degradation or by translational repression [13]. Brown and colleagues [14] have previously showed the ability of miRNA in controlling and restricting transgene expression to particular cell types. They used four perfectly complementary target sites for the cell line-specific miRNA at the 3′UTR of the transgene cassette to restrict transgene expression in all cell lines but the target one. This strategy was further used by the same group and others in a number of different cancer cell lines in order to show the broad applicability of this method [14-16]. mRNA degrade-ation and loss of translation often requires 100% complementarity between miRNA and its target [17]. However, it has been shown that the inhibition of the target mRNA is directly affected by the amount of miRNA within a cell and miRNA expression must reach a threshold in order to obtain a significant level of suppression [18].

The suppressive role of miRNA-143 in colorectal cancer, which is the third most frequently occurring tumor worldwide, is well established [19, 20]. It has been previously shown that the expression of miRNA-143 in transformed cells is severely decreased [21]. Such decrease in the cellular levels of miRNA-143 provides a good chance for cell-specific expression of a suicide gene. In normal cells, high levels of miR-143 prevent the expression of the suicide gene construct, while in transformed cells the absence of miRNA-143 can result in efficient expression of the suicide construct.

In a previous study, we showed that 3′UTR of Poliovirus in a reporter gene construct has enhancing effects on the stability of the corresponding mRN [22]. The present study, a Picornaviridae-like RNA suicide gene structure containing the internal ribosomal entry site of encephalomyocarditis virus and 3′UTR of Poliovirus was constructed. This construct was used to evaluate the effect of three tandem repeats of a complete complementary miRNA-143 target sequence on the expression of HSV1-TK and enhanced green fluorescent protein (EGFP) construct in SW480 cell line.

## MATERIALS AND METHODS

**Fig. 1:**
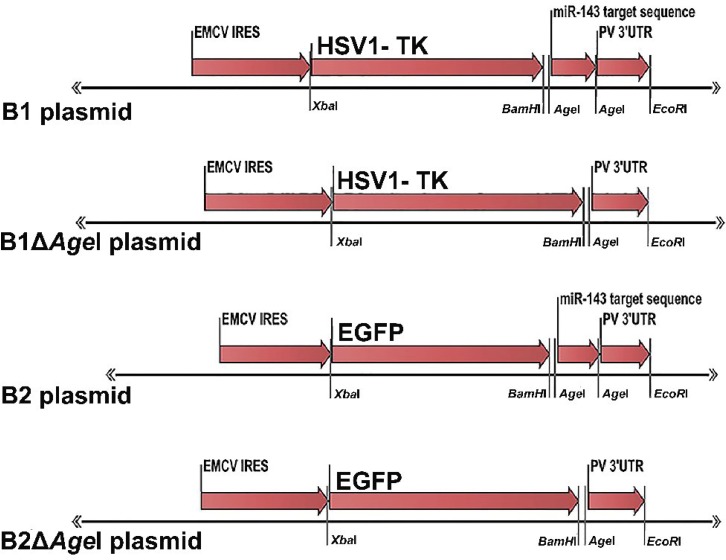
Schematic representation of constructed plasmids. B1 plasmid was chemically synthesized. Tandem repeat of hsa-miR-143 was removed by AgeI restriction enzyme to have B1ΔAgeI plasmid. HSV1-TK sequence was then replaced by EGFP to attain B2 plasmid. Finally, tandem repeat of hsa-miR-143 was removed by AgeI enzyme, and B2ΔAgeI was produced. EGFP, enhanced green fluorescent protein

***Plasmid design and construction.*** Plasmid B1 was chemically synthesized by Biomatik (Cambridge, Ontario, Canada). In this plasmid, the sequences of internal ribosomal entry site from encephalo-myocarditis virus (GenBank: X74312.1, nucleotides 285-868), HSV1-TK (KOS strain, GenBank: JQ673480.1, nucleotides 46613- 47743), three tandem repeats of complementary sequence for hsa-miR-143 (MI0000459) and 3′UTR from Poliovirus (Sabin 1 strain, GenBank: AY184219, nucleotides 7376–7441) were designed in tandem and subcloned into pcDNA^TM^3.1 (-). B1 plasmid was used as the basis for the construction of plasmid B1Δ*Age*I in which the tandem repeat of hsa-miR-143 was omitted by *Age*I enzyme digestion. For construction of B2 plasmid, EGFP open reading frame was amplified by sequence-specific primers (Table 1) from *pIRES2-EGFP* *plasmid* (Clontech Laboratories, Mountain View, CA) as the template. PCR thermal cycles were as follow: the initial denaturation at 95°C for 5 minutes and 30 cycles of 95°C for 30 seconds, 60°C for 20 seconds and 72°C for 90 seconds and a final expansion in 72°C for 5 minutes. Thymidine kinase sequence was then replaced by EGFP open reading frame after digestion with *Xba*I and *Bam*HI enzymes. B2Δ*Age*I plasmid was prepared by omitting the target sequence of hsa-miR-143 from B2 plasmid by *Age*I enzyme digestion procedure (Fig. 1). Plasmids were introduced to competent bacterial host using standard methods. All restriction enzymes used in this study were purchased from Thermo Fisher Scientific (Waltham, MA, USA). 

**Table 1:** Sequence of primers used in PCR and RT-PCR procedures

**Primer name**	**Primer sequence**
IRES forward	5’***TCTAGA*** GCCCCTCTCCCTCCCCCC3’
EGFP reveres	5’***GGATCC ***CGCGGCCGCTTTACTTGT3’
Tk forward	TCCTCGAGTCATAGCGCGGGT
Tk reveres	ATCCATGGCTTCGTACCCCTGCA

Recognition sites for *XbaI* and *BamHI* enzymes are indicated as bold letters. EFGP, enhanced green fluorescent protein; Tk, thymidine kinase

***microRNA synthesis. ***The sequence of hsa-miR-143-3p (MI0000459) was extracted from miRBase database (http://www.mirbase.org/) and chemically synthesized by Metabion (Martinsried, Germany). Mature sequence of hsa-miR-143 was chemically modified by addition of a 5'-2'-O-Methyl (5'-2'Ome-UGAGAUGAAGCAC UGUAGCUC). An antisense sequence was also chemically synthesized with no modifications (GAGC UACAGUGCUUCAUCUCA). Sense and antisense strands were diluted to a final concentration of 50 μM in RNase free water in order to generate mature duplexes of hsa-miR-143. Thirty microliter of each RNA oligonucleotide solution and 15 μl of siRNA annealing buffer (Qiagen, Hilden, Germany) were mixed and then the mixture was incubated at 90°C for 1 minute and 37°C for 45 minutes. Aliquots were kept at -70°C and thawed right before each experiment. Mimic House Keeping Positive Control 2 (GAPDH [glyceraldehyde 3-phosphate dehydrogenase], Thermo, Waltham, MA, USA) was used as a positive control. According to the manufacturer’s report, Mimic House Keeping Positive Control 2 (GAPDH) targets the 3′UTR of GAPDH mRNA and reduces the GAPDH mRNA levels by 90%. miRIDIAN miRNA Mimic Negative Control (Thermo, Waltham, MA) was used as negative control for miRNA. Manufacturer claims that this scrambled miR does not target any mRNA 3′UTR in human cell. 

***Cell culture.*** SW480 (a human colorectal adeno-carcinoma cell line) was obtained from National Cell Bank of Iran (NCBI, Pasteur Institute of Iran, Tehran, Iran*). *Cells were cultured in high glucose DMEM supplemented with 10% FBS (PAA, Pasching, Austria), 2 mM glutamine (PAA), and antibiotic-antimycotic solution (10,000 units of penicillin, 10,000 μg of streptomycin, and 25 μg of amphotericin B per mL) (PAA). Cells were maintained at 37ºC in a humidified incubator supplemented with 5% CO_2_. The flask media were changed every three days. 

***Plasmid linearization and in vitro transcription. ***All four plasmids were digested by *Eco*RI restriction enzyme to produce linearized plasmid DNA as templates for *in vitro* transcription. Also, the linearized plasmids were visualized by agarose gel electro-phoresis to avoid any contamination by the remaining undigested plasmids and to confirm the integrity of the linearized plasmid. These linearized plasmids were further purified by a standard *Phenol-chloroform extraction* to a final concentration of 1 µg/µL [23]. Only gel extracts with A_260/280 _ratio in the range of 1.8-2.0 were used to proceed into *in vitro* transcription. RNA transcripts were prepared by *in vitro* transcription by mMessage mMachine Kit (Ambion, Austin, TX, USA) according to the manufacturer’s recommendations. Purified linearized plasmid DNA was used as template. *In vitro* transcripts were treated with RNase-free DNase (Ambion, USA) at 37°C for 30 minutes to remove the possible DNA contamination. Then poly-A tail was added to the transcripts by Poly(A) Tailing Kit (Ambion) according to the manufacturer’s manual. Finally, mRNA was purified by a standard acidic phenol/chloroform extraction protocol followed by isopropanol precipitation [23]. Quantity of *in vitro* transcripts were evaluated by measuring the absorbance at 260 nM using Picodrop (Hinxton, UK). The ratio of 260-280 nM was used as an indicator of RNA quality. Only the* in vitro *transcripts with absorbance ratio at A_260/280 _between 1.9- 2 were used for transfection. The proportion of full-length transcripts was checked by formaldehyde denaturing agarose gel electrophoresis [23].

***mRNA and miR transfection. ***A previous study showed that the highest rate of pDNA-transfected SW480 cells was obtained by Effecten® transfection reagent (Qiagen, Germany) [24]. According to those results, Effecten® was used for the transfection of SW480 with *in vitro* transcribed mRNA. Several proportions of RNA: transfection reagent were tried out in order to obtain higher transfection rates. Briefly, the day before transfection, 50 × 10^3^ and 25 × 10^3^ cells were seeded in 24- and 48-well cell culture plates, respectively to achieve 80% confluency at the day of transfection. At the proper confluency, 1 or 2 µg of purified mRNA was used to transfect each well in 24- and 48-well plates, respectively at the ratio of 4:10 of RNA:Effecten®. Transfected cells were maintained at 37°C in a humidified incubator supplemented with 5% CO_2_. To confirm the functionality of miR processing machinery in SW480 and to show the efficacy of miR transfection by Effecten™ transfection reagent (miR:Effecten), we utilized different concentrations of Mimic House Keeping Positive Control 2 (GAPDH) to transfect cells at 80% confluency. miRIDIAN miRNA Mimic Negative Control was used to transiently transfect cells and was also used as scrambled negative control through the study. SW480 cells were transiently transfected with hsa-miR-143 duplexes 24 hours before transfecting cells with each mRNA when the confluency of cells was 80% [25]. 

***EGFP expression assay.*** Twenty four hours after transfection with EGFP expressing mRNA (or 48 hours after transfection with hsa-miR-143 duplexes), cells were collected by trypsinization. Trypsin was then neutralized by 0.1 mL of DMEM supplemented with 5% FBS. EGFP expression was evaluated by a flow cytometry instrument (CyFlowTM, Partec GmbH, Munster, Germany) equipped with FloMax@ software (Quantom Analysis GmbH, version 2.7). Results were acquired when forward scatter and side scatter channels were set in a linear mode and FL1 channel in a log mode. Events were triggered based on forward scatter. Gate was then set up based on the forward scatter-side scatter plot to avoid cellular debris. Mean fluorescence intensity of FL1 in the transfected cells and percentage of EGFP-expressing cells were the important parameters for comparison between groups. Cultured cells were visualized by direct fluorescence microscopy (INVERSO TC100 Epi Fluor, Cetti, France) prior to flow cytometry analysis. 

***RNA extraction and RT-PCR.*** One day after transfecting SW480 (50 × 10^3 ^cells per well) with *m*RNA, total cellular RNA was isolated by RNeasy kit (Qiagen, USA) according to the manufacturer’s manual. The quality and quantity of extracted RNA were assessed by measuring the absorbance in 260 and 280 nM. RT-PCR analysis by sequence-specific primers for thymidine kinase [24] was performed on 2 ng of purified RNA in 20 μL reactions using Power SYBR^®^Green RNA-to-CT™1-Step kit (Applied Biosystems, USA). Final abundance of mRNA was obtained by normalization to GAPDH mRNA as the reference gene. Relative abundance of mRNA was then calculated by ΔΔCT method, followed by further calculation through 2^-(ΔΔ(C^_T_^)) ^ method [26]. All real-time PCR analyses were carried out using Applied Biosystems7500Fast apparatus (Applied Biosystems, CA, USA). Standard GAPDH primers were purchased from NanoCinna Technologists (Tehran, Iran). All real-time analyses were carried out in triplicate.

***Cell proliferation assay.*** SW480 was seeded in a 48-well cell culture plate (25 × 10^3^ cells per well). Two days after transfection with miR, or one day after transfection with mRNA, different concentrations of GCV (0, 20, 40 and mg/ml) was added to the culture medium. Forty eight hours later, cell proliferation assay was performed by Cell Proliferation Kit II (XTT, Roche, Basel, Switzerland) according to the manufacturer’s manual. Four hours after incubation in CO_2_ incubator, the optical density of the plate was measured by a Synergy 4 Microplate Reader (BioTek, Winooski, VT) at 500 nM (690 nM as background). 

***Statistical analysis.*** Data were analyzed with the SPSS statistical package (SPSS for Windows, Version 16.0. Chicago, SPSS Inc.). All experiments were performed in at least triplicates. In addition, ANOVA test (when measuring differences among multiple means) was performed using SPSS in order to determine the significance of differences between means. Difference between means was considered statistically significant when* P *value was less than 0.05.

**Table 2:** Several ratios of RNA:Effectin and transfection efficiency calculated by flow cytometry analysis.

**Amount of RNA (μg)**	**Transfected cells at RNA:Effectin** ^TM^ ** ratio (%)**		
	**1:10**	**1:5**	**4:10**
0.5	3.80 ± 0.20	3.47 ± 0.02	-
1	22.50 ± 0.08	10.37 ± 0.01	-
2	-	-	54.54 ± 4.70
4	-	-	5.28 ± 0.24

The highest transfection percentages were obtained at 4:10 ratio (mean ± SD).

## RESULTS

***Sequencing and restriction mapping for the plasmid constructs. ***Results of the restriction enzyme analysis by *Hind*III and *Sal*I enzymes and DNA sequencing confirmed the accuracy of all four plasmids (data not shown). The correct cloning of procedure was confirmed by DNA sequencing.

**Fig. 2:**
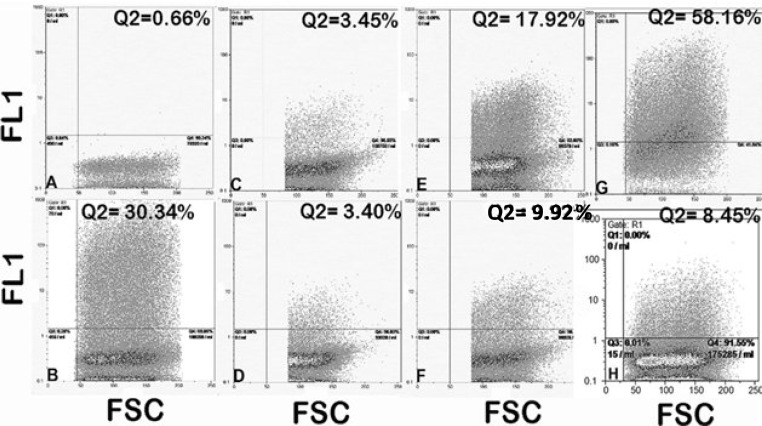
. Representative of flow cytometry graphs for SW480 transfected with different proportions of B2 mRNA: Effecten. (A) Untransfected SW480; (B) SW480 transfected with pEGFPN1 plasmid as a control for transfection procedure; (C) SW480 transfected with 0.5 µg B2 mRNA by 1:10 proportion to Effecten; (D) SW480 transfected with 0.5 µg B2 mRNA by 1:5 proportions to Effecten; (E) SW480 transfected with 1 µg B2 mRNA by 1:10 proportion to Effecten; (F) SW480 transfected with 1µg of B2 mRNA by 1:5 proportion to Effecten; (G) SW480 transfected with 2 µg B2 mRNA by 4:10 proportion to Effecten; (H) SW480 transfected with 4 µg B2 mRNA by 4:10 proportion to Effecten. In each graph, the emission wave lengths obtained from FL1 channel was plotted against forward-scattered light (FSC).

***Optimization of RNA:Effecten ratio. ***To achieve optimal transfection efficiency for RNA:Effecten combination, we selected several proportions between *in vitro* transcribed B2 RNA and Effecten™ (Table 2). Flow cytometry results showed that the highest transfection rates were achieved with RNA:Effecten ratio of 4:10. Therefore in further RNA transfection tests, this ratio of RNA:Effecten was used (Fig. 2). 

**Fig. 3:**
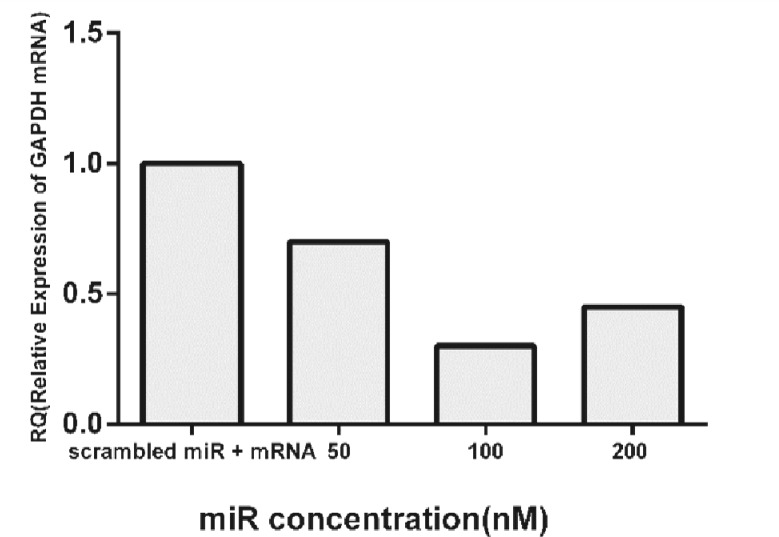
Relative expression (RQ) of GAPDH mRNA in SW480 transfected with different concentrations of Mimic House Keeping Positive Control 2 (GAPDH). Three different concentrations (50, 100, and 200 nM) of Mimic House Keeping Positive Control 2 (GAPDH) were used to attain the highest level of GAPDH mRNA knock down. Best results were observed when 100 nM of Mimic House Keeping Positive Control 2 (GAPDH) was used to transfect cells by the 4:10 ratio of miR:Effecten transfection reagent.

***Efficient knock down of GAPDH mRNA by Mimic House Keeping Positive control. ***The best ratio of RNA:Effecten was used to transfect SW480 with Mimic House Keeping Positive Control 2 (GAPDH) and miRIDIAN miRNA Mimic Negative Control. RT-PCR results indicated that the application of 100 nM of Mimic House Keeping Positive Control 2 (GAPDH) reduced the relative abundance of GAPDH mRNA by 75%. Application of 50 nM of Mimic House Keeping Positive Control 2 (GAPDH) reduced the levels of GAPDH mRNA by only by 30%. A higher concentration of mimic miR (200 nM) did not result in lower abundance level for GAPDH mRNA (Fig. 3). 

**Fig. 4:**
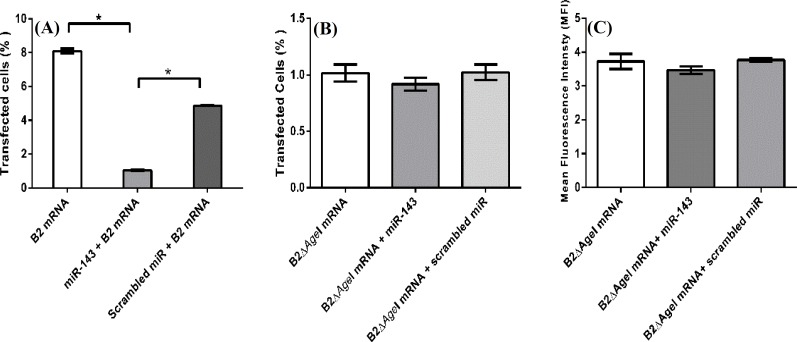
Effect of transient expression of hsa-miR-143 duplex on EGFP expression from B2 and B2ΔAgeI mRNA. EGFP expression in cells transfected with B2 mRNA, and hsa-miR-143 was reduced 88% and 80% in cells transfected with no microRNA and scrambled miR, respectively (A). Flow cytometry analysis of SW480 transfected with B2ΔAgeI mRNA showed that neither percentage of transfected cells (depicted as fold changes [B]) nor mean fluorescence intensity (C) changed in the presence of mimic miR and miR-143. * indicates the statistical significance (P < 0.05).

***EGFP expression was down regulated by hsa-miR-143. ***The expression of hsa-miR-143 is demolished in SW480 cell line [19]. Therefore, SW480 was used as the host cell line for a gain-of-function study. According to the results from transfection of SW480 with Mimic House Keeping Positive Control 2 (GAPDH), cells were transfected with 100 nM hsa- miR-143 duplex. As shown in Figure 4A, flow cytometry data showed that when hsa-miR-143 level was restored, EGFP expression was dropped by 88%. Similarly, when cells were transfected with B2 mRNA and scrambled miR, EGFP expression showed 80% reduction compared to the cells transfected with B2 mRNA and hsa-miR 143. When the target sequence of hsa-miR-143 was omitted from the mRNA construct in B2Δ*Age*I mRNA, results from the flow cytometry analysis showed a non-significant difference (*P *> 0.05) in the transgene expressing cells and the mean fluorescence intensity levels in the presence of hsa-miR-143 (Fig. 4B and 4C).

**Fig. 5:**
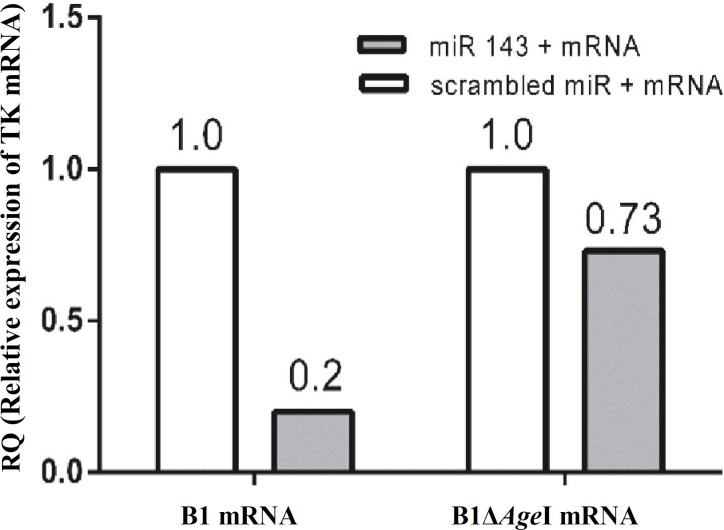
Relative abundance of B1 and B1Δ*Age*I mRNA in SW480 supplemented with hsa-miR-143 mimic. RT-PCR analysis revealed that the abundance of B1 mRNA was reduced by 80% in the presence of mimic miR. The reduction was less significant in the case of B1Δ*Age*I mRNA (28%).

***Down regulation of thymidine kinase mRNA in the presence of hsa-miR-143 in SW480. ***Results from RT-PCR analysis showed that hsa-miR-143 duplex can successfully reduce the abundance of B1 mRNA by 80% compared to control group that received scrambled miR and B1 mRNA. On the other hand, by omitting the target sequence for hsa-miR-143 in B1Δ*Age*I mRNA, the level of this mRNA was dropped only 28% when hsa-miR-143 was used to treat cells instead of scrambled miR (Fig. 5).

**Fig. 6:**
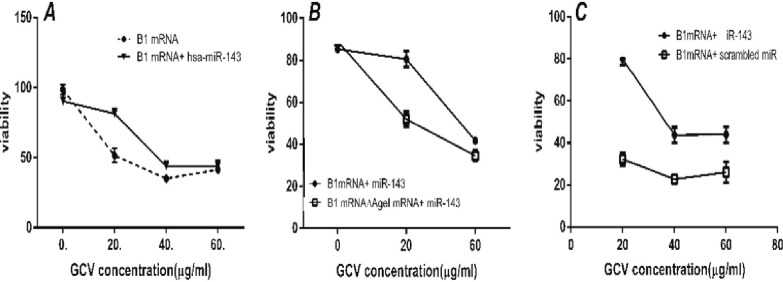
**. **Effect of mimic miR-143 and scrambled miR on inhibition of ganciclovir (GCV) cytotoxicity. (A) SW480 viability was evaluated after transfection with B1 mRNA and after treatment with different concentrations of GCV in the presence/absence of mimic miR-143. (B) SW480 viability after transfection with B1Δ*Age*I mRNA in the presence/absence of hsa-miR-143. Cell viability was decreased with increased GCV concentration in transfected cells. (C) SW480 viability was evaluated after transfection with B1 mRNA and after treatment with different concentrations of GCV in the presence of either mimic miR-143 or scrambled miR.

***More resistance to the cytotoxic effect of GCV in the presence of miRNA 143. ***It has been previously shown that concentrations between 20 to 80 µg/ml of GCV do not have cytotoxic effects on SW480 [27]. Analysis of variance for XTT results showed a statistically significant difference in SW480 viability when 20 µg/ml or higher concentrations, of GCV were used to treat cells transfected with B1 mRNA (*P *< 0.05). When 100 nM hsa-miR-143 was applied to a similar set of GCV concentrations followed by transfection with B1 mRNA, analysis of variance showed that difference in absorbance between wells treated with 0 and 20 µg/ml GCV was not statistically significant (*P* = 0.75). This observation also applied to the wells treated with 40 and 60 µg/ml GCV (*P* = 0.969) (Fig. 6A). It can be concluded that hsa-miR-143 has increased SW480 resistance to GCV. To prove that the resistance to GCV in the presence of hsa-miR-143 was due to sequence-specific interaction of miR-target sequence on B1 mRNA and hsa-miR-143, we evaluated the cytotoxicity of B1Δ*Age*I mRNA in the presence of hsa-miR-143 and different concentrations of GCV. Statistical analysis showed a significant difference in cell toxicity when 20 µg/ml of GCV were applied to the cells transfected with either B1 or B1Δ*Age*I mRNA (*P* = 0.00) (Fig. 6B). This result indicated that omitting the target sequence for hsa-miR-143 increases the sensitivity of cells to GCV significantly. Sequence-specific effect of miR-143 was further verified statistically by proliferation assay of the cells, which had been transfected with B1 mRNA along with 100 nM of miRIDIAN miRNA Mimic Negative Control (*P *< 0.0001). Cytotoxicity assay results showed that cells receiving the scrambled miR instead of hsa-miR-143 were highly sensitive to GCV (Fig. 6C).

## DISCUSSION

Gene therapy, as a novel strategy, has broadened our horizons in cancer therapy. Yet, a downside to this approach is safety issue, including transgene integration into the host genome. Here, we have used RNA agents to express two transgenes (EGFP as a reporter model and HSV1-TK as a therapeutic gene) in a human cell line and investigated the efficiency of cytotoxicity induced by HSV1-TK.

In complete agreement with previous studies [28, 29], flow cytometry results showed that our RNA agent can efficiently express EGFP (Fig. 2). The well-established fact that RNA macromolecules translocate between cells [30] can explain the different pattern of EGFP expression in flow cytometry graphs (Fig. 2B vs. 2G). An efficient exchange of RNA molecules between cells could be therefore considered as a compensation mechanism for low efficiency of the nucleic acid delivery in gene therapy [31].

To decrease the chance of unspecific expression of HSV1-TK in normal cells, we used a tandem of three repeats with absolute complementarity to hsa-miR-143 as an arming strategy. 

Although a very recent finding by Chivukula *et al.* [32] have raised controversy around the cell-autonomous tumor suppressor role of miR-143, this miRNA has been repeatedly reported and commonly accepted as a tumor suppressor in colorectal malignancies.

Flow cytometry (Fig. 4A) and cytotoxicity assay (Fig. 6A) results showed that when hsa-miR-143 was restored in SW480, the transgene expression by B1 and B2 mRNA was successfully deteriorated. When the abundance of hsa-miR-143 was restored in SW480, cytotoxicity of GCV was more strongly inhibited at low dose (20 µ/ml) by miR mimic. This result could be partly due to intrinsic sensitivity of SW480 to GCV at higher doses. When the target sequence for miR was omitted from the RNA construct, and the scrambled miR was used instead of miR mimic, RNA construct expressing HSV1-TK gene was strongly cytotoxic (Fig. 6). Similarly, EGFP expression level in the presence of miR-143 mimic was significantly restored when hsa-miR-143 target sequence was omitted from the construct (Fig. 4).

The comparison of the induced down-regulation of target mRNA by either a commercial duplex miR (Mimic House Keeping Positive Control 2 [GAPDH]) or our mimic duplex led us to believe that despite the differences in our design, efficient repression of translation could be achieved. Authors believe that this phenomenon could be partially due to the absolute complementarity of mimic miR and its antisense target sequence at the 3′UTR of the target mRNA. 

It is known that the specificity of the interaction of a miR and its target mRNA is the key issue in miR function and the degree of translation inhibition [33]. A previous study showed that for efficient endonuclease cleavage, base pairing at the site of cleavage (between bases 10 and 11) is require, and mRNA cleavage by endonucleases is usually favored when perfect base pairing between the miRNA and mRNA happens [34]. In accordance with findings of Yekta *et al. *[34], RT-PCR analysis of B1 mRNA showed a significant decrease in mRNA abundance in the presence of miR mimic (Fig. 5).

Based on the findings in the current research, we suggest that stabilized RNA constructs are convenient vehicles to deliver genetic information into recipient cells. Also, it can be suggested that due to aberrant miR expression signature of tumors, applying miR target sequences can be used as an effective arming strategy for tumor-specific expression of suicide genes. 
